# Superhard and Superconducting Bilayer Borophene

**DOI:** 10.3390/ma17091967

**Published:** 2024-04-24

**Authors:** Chengyong Zhong, Minglei Sun, Tariq Altalhi, Boris I. Yakobson

**Affiliations:** 1 College of Physics and Electronic Engineering, Chongqing Normal University, Chongqing 401331, China; zhongcy90@126.com; 2 Department of Materials Science and Nanoengineering, Rice University, Houston, TX 77005, USA; 3 Chemistry Department, Taif University, Taif 21974, Saudi Arabia; ta.altalhi@tu.edu.sa; 4 Department of Chemistry, Rice University, Houston, TX 77005, USA

**Keywords:** bilayer borophene, superhard, anisotropic superconductivity, electron–phonon coupling, strain effect, first-principles calculations

## Abstract

Two-dimensional superconductors, especially the covalent metals such as borophene, have received significant attention due to their new fundamental physics, as well as potential applications. Furthermore, the bilayer borophene has recently ignited interest due to its high stability and versatile properties. Here, the mechanical and superconducting properties of bilayer-$\delta_{6}$ borophene are explored by means of first-principles computations and anisotropic Migdal–Eliashberg analytics. We find that the coexistence of strong covalent bonds and delocalized metallic bonds endows this structure with remarkable mechanical properties (maximum 2D-Young’s modulus of ~570 N/m) and superconductivity with a critical temperature of ~20 K. Moreover, the superconducting critical temperature of this structure can be further boosted to ~46 K by applied strain, which is the highest value known among all borophenes or two-dimensional elemental materials.

## 1. Introduction

Superconductivity in two-dimensional (2D) materials has attracted perennial and ever-increasing attention over past decades, owing both to fundamental scientific interest and tantalizing applications [1,2,3,4,5]. One of the most important goals in superconductivity research is raising the superconducting transition temperature, T_c_. In general, superconducting materials can be classified into two categories: low-temperature superconductors, characterized by critical temperatures (T_c_) below 30 K, such as Nb-Ge films [6], and high-temperature superconductors with T_c_ exceeding 30 K, exemplified by Cu-based oxides [7,8,9]. Within the framework of the conventional Bardeen–Cooper–Schrieffer (BCS) theory [10], it is reasonably anticipated that metals composed of light elements have a better chance to induce a high T_c_, because the Debye temperatures within such metals are usually high enough to trigger a strong phonon-mediated superconducting pairing. More specifically, according to the celebrated McMillan–Allen–Dynes (MAD) formula [11,12]:

(1) $$T_{c}=\frac{\omega_{log}\;}{1.20}exp[\frac{-1.04(1+\lambda )}{\lambda -\mu^{*}(1+0.62\lambda )}]$$

T_c_ should be elevated by increasing the log-averaged characteristic phonon frequency ($\omega_{log}$) and the electron–phonon coupling (EPC) parameter, λ. The light elemental materials typically have high frequency phonon modes, enlarging $\omega_{log}$ and thus increasing T_c_. Furthermore, large phonon frequency and EPC potential $V=\frac{\lambda }{N\left(E_{f}\right)}$ induced by strong covalent bonding in light elemental material, together with $N\left(E_{f}\right),$ and the electronic density of states (DOS) at the Fermi level [13], should result in a higher T_c_. In fact, the metals with strong covalent bonding can be grouped as “covalent metals” [13] and their potential to harbor a high T_c_ in 2D form have been confirmed, e.g., hydrogenated monolayer MgB_2_ (67 K) [14], doped graphene (8.1 K~30 K) [15,16,17], hydrogenated monolayer borophene (32.4 K) [5] and especially 2D metal borides (1.4 K~72 K) [18,19,20,21,22].

Among covalent metals, 2D boron sheets (borophenes) recently came to the fore of superconducting research, motivated by their inherent metallicity, light weight and significant experimental progress on their synthesis [23,24,25]. Based on the first-principles calculations and MAD formula, Penev et al. [26] reported early on the T_c_ of the (experimentally synthesized $\delta_{6}$, $\beta_{12}$ and $\chi_{3}$ borophenes) to be in the range of 11.5 K~20.5 K and Gao et al. [27] found them to span 18.7 K–24.7 K. Using a more accurate and sophisticated anisotropic Migdal–Eliashberg (ME) equation, Zhao et al. [28] found the T_c_ in those borophenes in 26.2 K–33 K. Apart from intrinsic metallicity and light weight, another impetus for searching high-temperature superconductivity in borophenes is their vast polymorphs and superior mechanical properties, which provide a great benefit to potential superconductors, suggesting effective ways to modulate the superconductivity [29].

The monolayer borophenes have been intensively studied; however, the investigations are scarce [30,31,32] for bilayer or multilayer borophenes, where more intriguing properties and tunability could be manifested, compared to their monolayer counterparts. Very recently, the realization of bilayer borophenes were reported [33,34]. Unfortunately, the first reported bilayer α-borophene is expected to be unstable if peeled off from the metal substrate [35], and the $\beta_{12}$-like bilayer synthesized on Cu(111) surface appears complicated due to ambiguity (with more than three hundred atoms in its unit cell and the atomic structure still in debate), preventing an exploration of its properties or further application. It can be anticipated that more stable and versatile bilayer borophenes are still to be unveiled for research.

**Figure 1:**
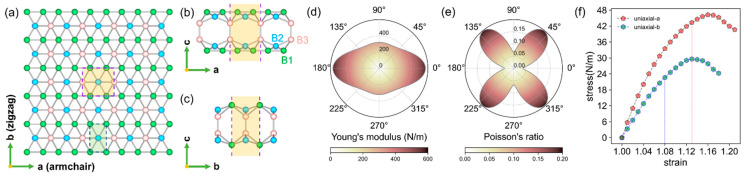
(**a**) Planar and (**b**,**c**) side views of the atomic structure of BL-δ_6_. The yellow shaded region is its unit cell, and the green shaded region is the unit cell of δ_6_. There are three irreducible boron atoms colored as B1 (green), B2 (blue), and B3 (pink). The direction dependence of (**d**) Young’s modulus and (**e**) Poisson’s ratio of BL-δ_6_. (**f**) Stress–strain curves of BL-δ_6_, the vertical blue and red dashed lines denote the fractured stress along the *a* and *b* directions, respectively.

In this work, we investigate the mechanical and superconducting properties of a bilayer composed of two covalently bonded $\delta_{6}$ borophene monolayers, “(BL)-$\delta_{6}$” in Figure 1a–c. The compact atomic structure endows BL-$\delta_{6}$ with remarkable stability and tantalizing properties compared to its monolayer counterparts. Its computed high Young’s modulus of 570 N/m is even higher than 346 N/m for graphene (or near 280 N/m “per layer”, for a fair comparison). Based on the anisotropic ME equation, we find its T_c_ = 20 K and can be boosted to 46 K by strain effect, reaching the highest T_c_ found among all borophenes or elemental 2D materials known to date. The coexistence of superhard (in basal plane direction) and superconducting properties in one material is rare, making BL-$\delta_{6}$ a fascinating superconductor for emergent nanoscale devices such as quantum interferometers, superconducting transistors, superconducting qubits, and wear-resistant parts of superconducting devices [3,36,37].

## 2. Results and Discussion

### 2.1. Atomic Structure and Mechanical Properties

BL-$\delta_{6}$ and $\delta_{6}$ share the same top view (Figure 1a), and BL-$\delta_{6}$ can be viewed as AB stacking of two $\delta_{6}$ monolayers bonded via interlayer covalent bonds (side view in Figure 1b,c). The unit cell of BL-$\delta_{6}$ is a rectangle with lattice constants *a* = 3.243 Å, *b* = 2.883 Å (Table 1), which is obtained with a Vienna ab initio Simulation Package [38,39] (computational details can be found in Supplementary Note S1). The total energy of BL-$\delta_{6}$ is computed to be lower than all experimental synthesized monolayers, due to the interlayer bonding: −6.38 eV/atom, which is 0.171 eV/atom, 0.125 eV/atom and 0.113 eV/atom lower than that of the synthesized $\delta_{6}$, $\beta_{12}$ and $\chi_{3}$, respectively (see Table 1, for atomic structures see Figure S2). Moreover, the slight dynamical instability of $\delta_{6}$ is also eliminated by the interlayer bonding of BL-$\delta_{6}$. The energetic stability of BL-$\delta_{6}$ was also confirmed by an ab initio evolutionary global structure search in our prior work [40] and reported by Zhou et al. [41]. Given that the monolayer $\delta_{6}$ has been fabricated on a Ag(111) substrate, the thermal stability of BL-$\delta_{6}$ is also tested on Ag(111) substrate by an AIMD simulation at 500 K, and one can observe that its whole structure is well maintained after 5 ps with a timestep of 1 fs under such a high temperature (Figure S3). Therefore, it is well-expected that BL-$\delta_{6}$ will be accessed experimentally, supported by its outstanding energetic and thermal stability, and the progress in bilayer borophene synthesis [42,43].

We obtain the four independent elastic constants for BL-$\delta_{6}$: *C*_11_ = 570 N/m, *C*_22_ = 322 N/m, *C*_12_ = 22 N/m, and *C*_44_ = 146 N/m, which apparently satisfy the Born–Huang criteria [46]: $C_{11}C_{22}-C_{12}^{2}>0$ and $C_{44}>0$, demonstrating the mechanical stability of BL-$\delta_{6}$. The θ dependence of in-plane Young’s modulus *Y* and Poisson’s ratio ν of BL-$\delta_{6}$ are plotted with polar coordinates in Figure 1d,e (computational details can be found in Supplementary Note S2 and Figures S5 and S6). We find that the in-plane Young’s moduli and the Poisson’s ratios of BL-$\delta_{6}$ are highly anisotropic. For comparison, the values of elastic constants, Young’s moduli, Poisson’s ratios of $\delta_{6}$, $\beta_{12}$, $\chi_{3}$ and graphene are summarized in Table 1. The introduced B3-B3 bonds in BL-$\delta_{6}$ boost the *Y*_a_ and *Y*_b_ to 570 N/m and 321 N/m, respectively. Although graphene is well-known as the strongest 2D material, along the armchair direction *Y*_a_ = 570 N/m exceeds the *Y*_a_ = 364 N/m of graphene, partially due to the strong directional B-B σ-bonds, making it stand out among 2D materials. In contrast, the *Y*_b_ = 321 N/m (still comparable to graphene) is lower, due to the much weaker multicenter bonds involved in the zigzag direction.

We find the fracture strain is 13% along the *a* and 8% along the *b* direction, according to the strained phonon spectra (Figure S4), before reaching the elastic maximum of 16% and 14% (Figure 1f). Thus, the failure mechanism of BL-$\delta_{6}$ is phonon instability, for both directions, which is different from that of $\delta_{6}$ (elastic instability in the zigzag direction and phonon instability in the armchair direction) [47]. The fracture strengths of BL-$\delta_{6}$ are 43 and 23 N/m along the *a* and *b* directions, much above that of $\delta_{6}$ (20.26 and 12.98 N/m) [47], black phosphorene (9.99 and 4.44 N/m) [48], and even graphene (40.41 and 36.74 N/m) [49] along the zigzag direction. Considering the ultrahigh Young’s moduli and large fracture strength, one can judge the BL-$\delta_{6}$ being in-plane superhard.

### 2.2. Electronic Properties

**Figure 2:**
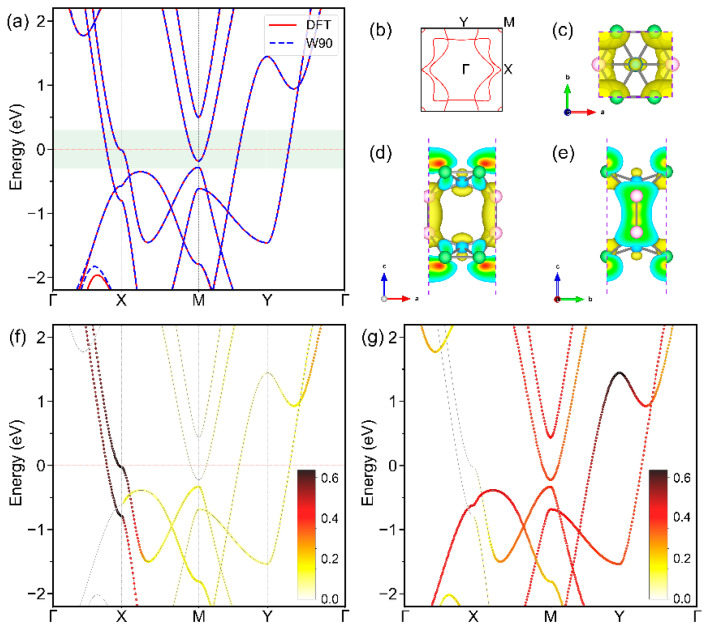
(**a**) Electronic band structures of BL-δ_6_ by DFT (red solid lines) and Wannier90 (blue dashed lines). (**b**) Fermi surface, (**c**–**e**) top and side views of the charge density corresponding to the energy range shaded green in (**a**), and electronic band structures weighted by the *p_y_* orbital of B1 and B3 atoms (**f**) and the *p_z_* orbital contributions of all boron atoms (**g**) of BL-δ_6_.

The electronic structure of BL-$\delta_{6}$ is summarized in Figure 2. Three bands cross the Fermi level and form the Fermi surface, of which one forms a small electron pocket centered around the corner of the first BZ and the other two contribute to the rest of Fermi surface (Figure 2b). The atomic- and orbital-resolved band structures suggest that the main contribution near the Fermi level is from the $p_{y}$ orbital of B1/B3 atoms and the *p_z_* orbital of all B atoms (Figure 2f,g, more details in Figure S7), which is also confirmed by the charge distribution in the states within ±0.3 eV from the Fermi level (Figure 2c–e). Using Wannier90 (v 3.1.0) code [50], the Wannier-interpolated band structures are also provided and show excellent agreement with those by first-principles calculations (Figure 2a), which lays a clear ground for subsequent anisotropic superconductivity calculations, as implemented in electron–phonon Wannier (EPW v 5.4) code [51].

### 2.3. Isotropic and Anisotropic Superconducting Properties

**Figure 3:**
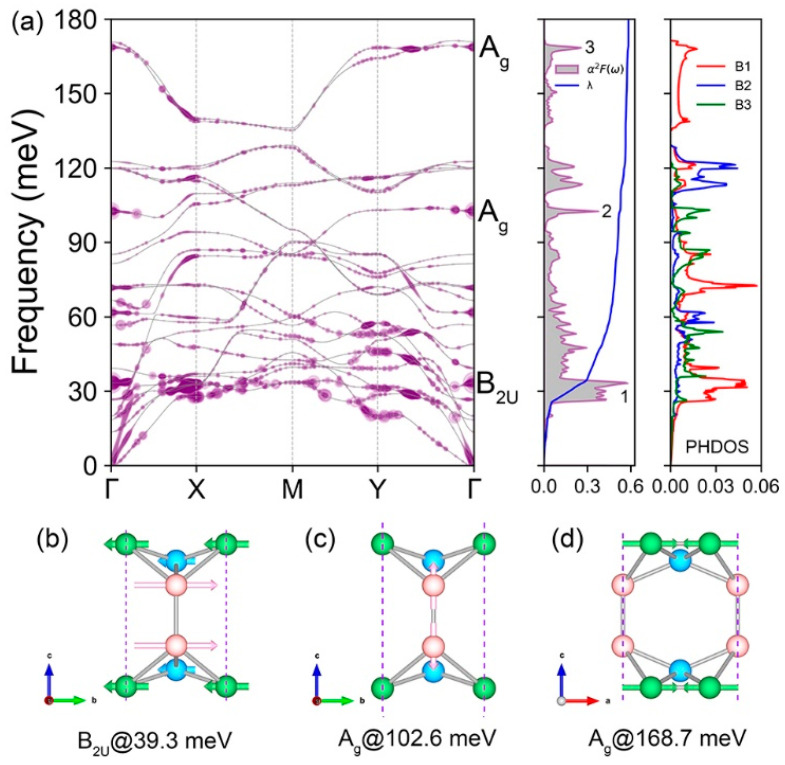
(**a**) From left to right: phonon band structure of BL-δ_6_ weighted by EPC *λ_qν_* with purple circles, isotropic Eliashberg function *α*^2^*F* and EPC *λ*(*ω*), and phonon density of states contributed by different kinds of boron atoms. (**b**–**d**) Vibrational modes of the B_2u_ mode and the two A_g_ modes at Γ, as labeled in (**a**).

In the phonon spectrum of BL-$\delta_{6}$ (Figure 3a), we find no negative frequencies, demonstrating its dynamic stability. This is in stark contrast to $\delta_{6}$, a layer whose phonon dispersion displays small imaginary frequency near the Γ point [24]. Mechanically intuitive, the BL-$\delta_{6}$ is stabilized by the covalent B3-B3 bonds. The (ν) mode- and (*q*) momentum-resolved EPC $\lambda_{q\nu }$ is also given, calculated by the following equation:

(2) $$\lambda_{q\nu }=\frac{2}{\hbar N\left(f\right)N_{k}}\sum_{nmk}\frac{1}{\omega_{q\nu }}{\left|g_{k,q\nu }^{nm}\right|}^{2}\delta \left(\epsilon_{k}^{n}\right)\delta (\epsilon_{k+q}^{m}),$$

where *N*_k_ represents the total number of *k* points in the *k* space, $g_{k,q\nu }^{nm}$ is an EPC matrix element, *n*/*m* and ν represent the indices of electronic bands and phonon mode, $\epsilon_{k}^{n}$ and $\epsilon_{k+q}^{m}$ are the eigenvalues with respect to the Fermi level, and $\omega_{q\nu }$ is the phonon frequency. We found three modes (B_2u_ and two A_g_) have comparatively large EPC near the Γ point (left panel of Figure 3a). In the B_2u_ shear mode at 39.3 meV, the B1 and B2 atoms move in one direction and the B3 atoms move in the opposite (Figure 3b). The first A_g_ mode at 102.6 meV corresponds to a bond stretching contributed by B3 atoms (Figure 3c). The second A_g_ mode at 168.7 meV is also a stretch mode, but predominantly contributed by B1 atoms (Figure 3d). The vibrational contribution of these three modes can also be inferred from the phonon density of states (right panel of Figure 3a). The Eliashberg spectral function $\alpha^{2}F(\omega )$ is a key quantity, which determines the total EPC λ by the following equation:

(3) $$\lambda =\sum_{q\nu }\lambda_{q\nu }=2\int \frac{\alpha^{2}F\left(\omega \right)}{\omega }d\omega$$

Accordingly, the $\alpha^{2}F(\omega )$ and cumulative EPC λ(ω) are calculated (middle panel of Figure 3a). We observe three sharp peaks mainly contributed by the three phonon modes analyzed above. Correspondingly, the EPC strength λ calculated by Equation (3) is 0.59 and the T_c_ of BL-$\delta_{6}$ within the MAD approximation calculated by Equation (1) is 10.1 K.

**Figure 4:**
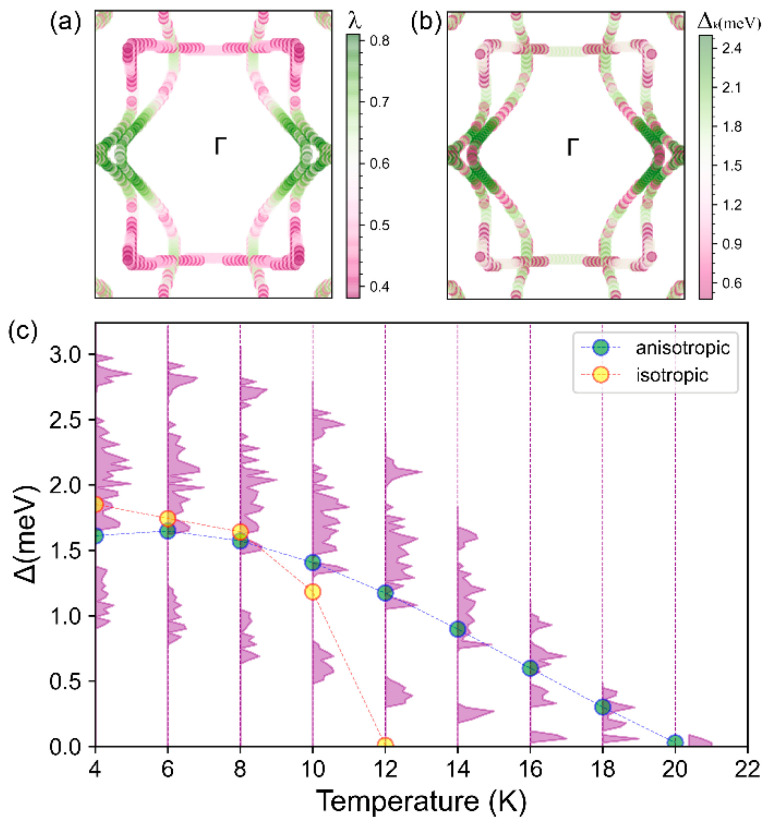
(**a**) Momentum-dependent EPC *λ_k_* across the full BZ and (**b**) momentum-dependent superconducting band gap ∆*_k_* at 10 K and projected onto the Fermi surface. (**c**) Variation in the superconducting gap ∆*_k_* with temperature, calculated by solving the ME equations in the isotropic approximation (yellow dots and dashed line interpolated) and with the fully anisotropic solution, where the purple shadowed regions indicate the magnitude distribution of the ∆*_k_* and the light green dots connected with the dashed line represents the average value of the entire anisotropic ∆*_k_*.

Akin to structure and mechanics anisotropy, the Fermi surface of BL-$\delta_{6}$, formed by multiple bands with different orbital contributions (*p*_y_ and *p*_z_ orbitals, Figure 2f,g) is also significantly anisotropic. For such a system with a complicated Fermi surface, using the anisotropic ME equation is essential, in order to obtain an anisotropic EPC, and an accurate Tc, compared with the isotropic superconductivity calculated from the MAD formula using an isotropic EPC [28]. As shown in Figure 4a,b, the variation in momentum-dependent EPC parameter $\lambda_{k}$ and the superconducting gap $\Delta_{k}$ at 10 K displays similar anisotropy, i.e., the maximum along the Γ−X direction and the minimum along the Γ−Y direction. We find the superconducting gap ratio $\Delta^{aniso}$ = ($\Delta^{max}-\Delta^{min})/\Delta^{ave}$ = (2.499 − 0.444)/1.470 ≈ 140%, a measure of its strong anisotropy at the Fermi surface, also signifying that the anisotropic ME formula is indispensable for predicting T_c_. Figure 4c shows the evolution of the superconducting gap as a function of temperature, based on the ME equations solved in either isotropic or fully anisotropic approximations. The T_c_ is identified as the lowest temperature at which the vanishing gap is observed. Under the isotropic approximation T_c_ = 12 K, comparable to the value (10 K) obtained by the MAD formula but is much lower than the value calculated with the fully anisotropic approximation (20 K), further attesting to the anisotropic superconducting nature of BL-$\delta_{6}$, omitted previously [32].

The mechanical robustness of BL-$\delta_{6}$ permits a consideration of whether the tensile strain could enhance the T_c_, motivated mainly by two reasons. First, according to Equation (2), it can be seen that EPC $\lambda_{q\nu }$ is inversely proportional to the phonon frequency $\omega_{q\nu }$, which would be helpful to lower, perhaps by tension, weakening the atomic force constants and thus softening the phonon modes. Second, the atomic orbitals overlap is reduced so that the electronic bands become less dispersive, enlarging the $N(E_{f})$, which provides more electrons susceptible to pairing interactions, mediated by dynamic phonons, to certainly contribute to the T_c_ rise.

**Figure 5:**
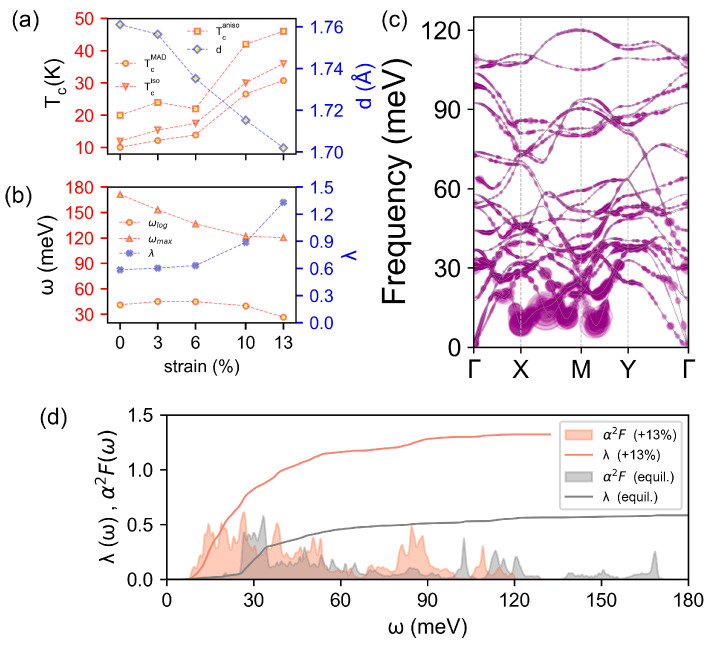
(**a**) Evolution with tensile strain along the *a* direction, of the *T*_c_ at three approximations (MAD, circles; isotropic ME, triangles; anisotropic ME, squares) and the distance between $\delta_{6}$ planes measured as B3-B3 bond length *d*. (**b**) Evolution of EPC *λ* (solid crosses), the log-weighted frequency *ω_log_*, (circles) and maximum phonon frequency *ω_max_* (triangles) versus the strain. (**c**) Phonon band structure weighted by EPC *ω_qν_* under 13% strain. (**d**) Isotropic Eliashberg *α*_2_*F* and EPC *λ* with 13% tensile strain along the *a* direction. Data with no strain are shown in gray, for comparison.

To examine the effect of strain on the BL-$\delta_{6}$ superconductivity, the evolution of EPC λ, the log and maximum frequencies $\omega_{log}$ and $\omega_{max}$, as well as T_c_, are all calculated within three approximations (i.e., MAD, isotropic ME, anisotropic ME), as a function of tensile strain along the *a* direction, and presented in Figure 5a,b (for details see Figures S8–S11). As expected, the increment in T_c_ is accompanied by a decrease in frequency-dependent $\omega_{log}$/$\omega_{max}$ and an increase in EPC λ (Figure 5b). The phonon spectrum under a 13% tension along *a* (near fracture strain) weighted by $\lambda_{q\nu }$, the Eliashberg spectral function $\alpha^{2}F(\omega )$ and EPC $\lambda \left(\omega \right)$ are shown in Figure 5c,d. The red shift of phonon frequency can be clearly observed and the enhanced EPC $\lambda_{q\nu }$ due to the phonon softening can be seen in all frequency ranges (Figure 5d). In addition, the contribution of low-frequency part becomes more dominant. In particular, four largely softened phonon Kohn anomalies with considerable EPCs around 15 meV appear on the path of X-M-Y. Eventually, these combined effects lead to a significant increase, by strain, in λ from 0.59 to 1.33 and T_c_ from 20 K to 46 K. It should be emphasized that 46 K is the highest T_c_ among all borophenes and elemental 2D materials (Table 2). It should be noted that for bilayer or multilayer materials with positive Poisson ratios, such as BL-$\delta_{6}$, the tensile strain would lead to vertical shrinking (Figure 5a), suggesting that the vertical pressure may also be an effective way to enhance the T_c_ of BL-$\delta_{6}$, as the tensile strain does.

Before concluding, a few remarks need to be addressed. Superhard materials are usually semiconductors with large bandgaps or insulators, because the majority of electrons are bound into covalent bonds, depleting any excess electrons from electron transport. For instance, diamond is an insulator and the hardest material known. It may seem that metallicity and superhardness cannot coexist in one covalent material. However, this dilemma can be resolved in 2D borophenes, because the electron deficiency nature of boron atom enables strong localized covalent bonds and delocalized multicenter metal bonds in one system, which endows the system with superb mechanical performance and excellent metallicity, as evidenced by the high Young’s moduli and metallicity in BL-$\delta_{6}$.

It is worth mentioning that research on BL borophenes is still in its infancy and has just been ignited by two recent independent experimental investigations. We would also like to point out that in addition to the striking mechanical properties and superconductivity studied in this work, BL-$\delta_{6}$ is expected to possess other interesting properties, such as anisotropic plasmonics [52] and ultrahigh thermal conductivity [53], awaiting more research. On the other hand, the excellent mechanical stability is also beneficial for BL-$\delta_{6}$ transfer to or even growth on some inert substrates [54], for convenience of characterization. Our results also show that the interlayer bonds, while strengthening the bilayer, do not destroy the σ-bond resonance nor the conducting π-bonds [55], adding a fine tuning-knob to the properties. This also highlights the potential of covalent metals in the quest for high T_c_ superconductors.

In our investigation, the tensile strain is applied to enhance the superconducting temperature of bilayer borophene. In addition to strain, the phenomenon of disorder, particularly correlated disorder, can also significantly enhance the superconductivity in a material [56,57]. In bilayer borophene, manipulating disorder could pave new paths to enhance superconducting performance. On the other hand, a caveat common for all ≤2D materials, due to the Mermin–Wagner theorem, must be mentioned, since it restricts the stability, both merely structural and of the superconducting phase. Nevertheless, in any BL borophene realization, the sample finite size (and support by a 3D-substrate) should mitigate these concerns, although quantifying the size limitation is far beyond the scope of the present study.

## 3. Conclusions

In conclusion, we comprehensively investigated mechanical and superconducting properties of a bilayer borophene (BL-$\delta_{6}$), which can be viewed as AB stacking, with interlayer covalent bonding, of earlier realized $\delta_{6}$ borophene. The original good stability of $\delta_{6}$ and the introduced covalent bond endows BL-$\delta_{6}$ with very prominent energy—thermodynamic, thermal, and mechanical stability, suggesting that it has a high chance of occurrence in experiments. The strong directional σ B-B bonds and very compact atomic configuration give BL-$\delta_{6}$ a 2D Young’s modulus along the *a* direction (569.4 N/m) higher than graphene (346.1 N/m), while ensuring that BL-$\delta_{6}$ can sustain an ultimate 13% and 8% tensile strain along the *a* and *b* directions, respectively. Furthermore, according to a fully anisotropic solution of the ME equation, we predict BL-$\delta_{6}$ is a conventional phonon-mediated 2D superconductor with T_c_ as high as 20 K. We also highlighted the effects of tensile strain on EPCs and found that the T_c_ can be boosted to 46 K at a 13% tensile strain along the *a* direction. To the best of our knowledge, it is the highest value currently known for borophene or elemental 2D materials. Our findings also consolidated the justification that covalent metals, such as borophene, should benefit the search for high T_c_ superconductors. The concurrent superior mechanical performance and excellent superconductivity is scarce; thus, promising BL-$\delta_{6}$ many potential applications, such as quantum interferometers, superconducting transistors, superconducting qubits, and even wear-resistant parts for superconducting devices.

## Figures and Tables

**Table 1:** The space group, lattice constants (Å), elastic constants (N/m), Young’s moduli (N/m), Poisson’s ratios, and cohesive energies (eV/atom) of BL-$\delta_{6}$, $\delta_{6}$, $\beta_{12}$, $\chi_{3}.$

System	Space Group	*a*	*b*	*C*_11_	*C*_22_	*C*_12_	*C*_44_	*Y*_a_	*Y*_b_	$v_{a}$	$v_{b}$	*E*_c_
$\mathrm{BL}-\delta_{6}$	Pmmm	3.243	2.883	570.0	322.0	22.0	146.0	569.4	321.4	0.038	0.068	−6.380
$\delta_{6}$	Pmmn	1.614	2.874	398.6	171.8	−4.0	93.9	398.6	171.8	−0.01	−0.02	−6.209
$\delta_{6}$ [24]		1.617	2.865	398.0	170.0	−7.0	94.0	398.0	170.0	−0.04	−0.02	
$\beta_{12}$	Pmmm	2.931	5.065	187.0	218.6	36.8	62.7	180.8	211.4	0.168	0.197	−6.255
$\beta_{12}$ [44]				185.5	210.5	37.0	68.5	179.0	203.1	0.176	0.199	
$\chi_{3}$	Cmmm	8.407	2.912	207.3	198.5	28.2	60.0	203.3	194.7	0.135	0.142	−6.267
$\chi_{3}$ [44]				201.0	185.0	21.5	60.5	198.5	182.7	0.116	0.107	
Graphene	P6/mmm	2.468		356.9		62.2	147.3	346.1		0.174		
Graphene [45]		2.470		352.7		60.9	145.9	342.2		0.173		

**Table 2:** The EPC *λ*, T_c_ calculated with MAD formula and anisotropic ME equation of δ_6_, β_12_, χ_3_, for Li-doped graphene [15] and BL-δ_6_.

System	λ	$T_{c}^{MAD}$ (*K*, $\mu^{*}=0.1$)	$T_{c}^{aniso}$ (*K*, $\mu^{*}=0.1$)
$\delta_{6}$ [26]	1.10	20.5	
$\delta_{6}$ [28]	0.82		27.0
$\beta_{12}$ [26]	0.80	16.1	
$\beta_{12}$ [27]	0.89	18.7	
$\beta_{12}$ [28]	1.01		33.0
$\chi_{3}$ [26]	0.60	11.5	
$\chi_{3}$ [27]	0.95	24.7	
$\chi_{3}$ [28]	0.79		26.2
Graphene (Li deposition) [15]	0.61	8.1	
$\mathrm{BL}-\delta_{6}$	0.59	10.1	20
$\mathrm{BL}-\delta_{6}$ [32]	0.61	11.9	
$\mathrm{BL}-\delta_{6}$ (13%)	1.33	30.78	46

## Data Availability

Data are contained within the article.

## Supplementary Materials

The following supporting information can be downloaded at: https://www.mdpi.com/article/10.3390/ma17091967/s1. Figure S1: The evolution of T_c_ with different *k* and *q* meshes. Figure S2: The atomic structures of (a) δ_6_, (b) β_12_ and (c) χ_3_. Figure S3: (a) The total potential energy fluctuation of BL-$\delta_{6}$ during AIMD simulation at 500 K. (b) The top view and (c) side view of the atomic configuration of BL-$\delta_{6}$ on Ag (111) surface after 5 ps of AIMD simulation at 500 K. Figure S4: The phonon spectra of strains under (a) 13% and (b) 14% along a axis, (c) 8% and (d) 9% along b axis. Figure S5: The strain energy per area under different kinds of strains for BL-$\delta_{6}$. Figure S6: The strain energy per area under different kinds of strains for (a) $\delta_{6}$, (b) $\beta_{12}$, (c) $\chi_{3}$ and (d) graphene. Figure S7: The atomic and orbital resolved band structures of BL-$\delta_{6.}$ Figure S8: (a) The electronic band properties of BL-$\delta_{6}$ under 3% tensile strain along a direction, red line and blue dashed line denote the results calculated from DFT and Wannier90, respectively. (b) Phonon band structure of BL-$\delta_{6}$ weighted by EPC $\lambda_{q\nu }$ with purple circles, isotropic Eliashberg function $\alpha^{2}F$ and EPC $\lambda \left(\omega \right)$, PHDOS from different kinds of boron atoms’ contribution. (c) Evolution of the superconducting gap $\Delta_{k}$ as a function of temperature, calculated by solving the ME equations in the isotropic approximation (yellow dots and dashed line interpolation) and with a fully anisotropic solution where the purple shadowed regions indicate the magnitude distribution of the $\Delta_{k}$ and the light green dots connected with dashed line represents the average value of the entire anisotropic $\Delta_{k}$. Figure S9: The results of BL-$\delta_{6}$ under 6% tensile strain along a direction. The meaning of (a–c) are the same as Figure S8. Figure S10: The results of BL-$\delta_{6}$ under 10% tensile strain along a direction. The meaning of (a–c) are the same as Figure S8. Figure S11: The results of BL-$\delta_{6}$ under 13% tensile strain along a direction. The meaning of (a–c) are the same as Figure S8. Refs. [38,39,50,51,58,59,60,61,62,63,64,65] are cited in the Supplementary Materials.
