# Supplementary material for: Superhard and Superconducting Bilayer Borophene
Source: Materials (Basel). 2024 Apr 24;17(9):1967. doi: 10.3390/ma17091967 (PMC11084974; doi:10.3390/ma17091967)
Supplement: Supplementary file 1 [file materials-17-01967-s001.zip › materials-2944761-supplementary.pdf]

Supporting Information

Superhard, superconducting bilayer borophene

Chengyong Zhong[1], Minglei Sun[2 *], Tariq Altalhi[3], and Boris Yakobson[3,4 *]

[1] College of Physics and Electronic Engineering, Chongqing Normal University, Chongqing 401331, China

[2] Department of Materials Science and Nanoengineering, Rice University, Houston, TX 77005, USA

[3] Chemistry Department, Taif University, Taif 21974, Saudi Arabia

[4] Department of Chemistry, Rice University, Houston, TX 77005, USA

Correspondence: *M.S., minglei.sun@rice.edu; *B.I.Y., biy@rice.edu

Supplementary note 1:

First-principles calculations were carried out within the Vienna *ab initio* Simulation Package (VASP)[1, 2] based on density functional theory (DFT). The exchange-correlation functional of Perdew-Burke-Ernzerhof (PBE)[3] along with the projector-augmented wave (PAW)[4] pseudopotentials were employed for the self-consistent total energy calculations and geometry optimization. The kinetic energy cutoffs were chosen to be 400 eV and 500 eV for boron and carbon atoms. Atomic positions were relaxed until the energy difference was small than 10^{-8} eV and the maximum Hellmann-Feynman forces imposed on any atoms were below 10^{-4} eV/Å. The Brillouin Zone (BZ) was sampled with a $10 \times 12 \times 1$ Monkhorst-Pack *k*-point mesh. The vacuum thick was set to 25 Å. The thermal stability was investigated with AIMD simulations in a canonical ensemble with a Nose-Hoover thermostat.[5, 6]

The phonon and superconducting properties were calculated in the Quantum-ESPRESSO (QE) package.[7] The PBE exchange-correlation functional and PAW pseudopotential with a 60 Ry cutoff energy were adopted to model the electron-ion interactions. The structural and electronic properties were recalculated by QE, and the results are in good agreement with those by VASP. The vibrational properties and phonon perturbation potentials were calculated on a $10 \times 10 \times 1$ mesh of q-points within the framework of density-functional perturbation theory,[8] combing with Marzari-Vanderbilt smearing scheme of width 0.03 Ry and $20 \times 20 \times 1$ k-point mesh. Once the phonon perturbation potentials were obtained in QE, we solve the ME equation both in the isotropic and anisotropic approximations by using the Electron-phonon Wannier (EPW) 5.4 code[9] to obtain the superconducting gap and its temperature evolution. Fine electron ($120 \times 120 \times 1$) and phonon ($60 \times 60 \times 1$) grids were used to interpolate the EPC constant through the maximally localized Wannier functions[10] as implemented in the EPW code. All the parameters are carefully tested to achieve the convergence (Figure S1). In all cases, a 0.8 eV cutoff for the Matsubara frequency was chosen; the Dirac delta functions for electrons and phonons were smeared out with the widths of 0.02 eV and 0.3 meV, respectively; and a typical value of 0.1 was used for the screened Coulomb parameter μ^*.

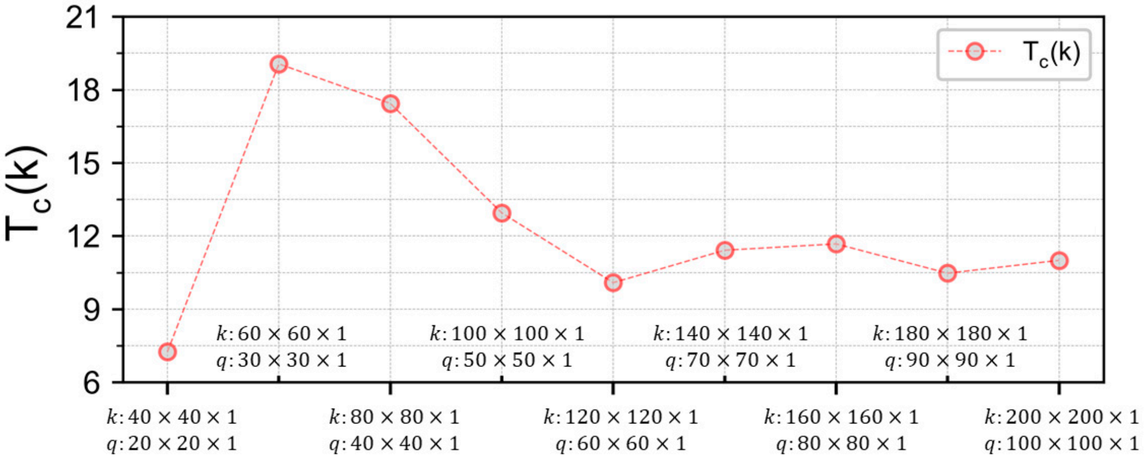

Figure S1. The evolution of Tc with different *k* and *q* meshes.

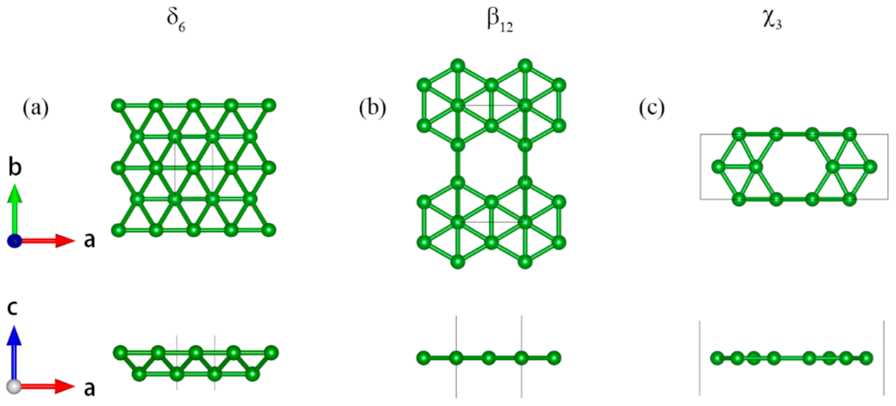

Figure S2. The atomic structures of (a) δ_6, (b) β_{12} and (c) χ_3.

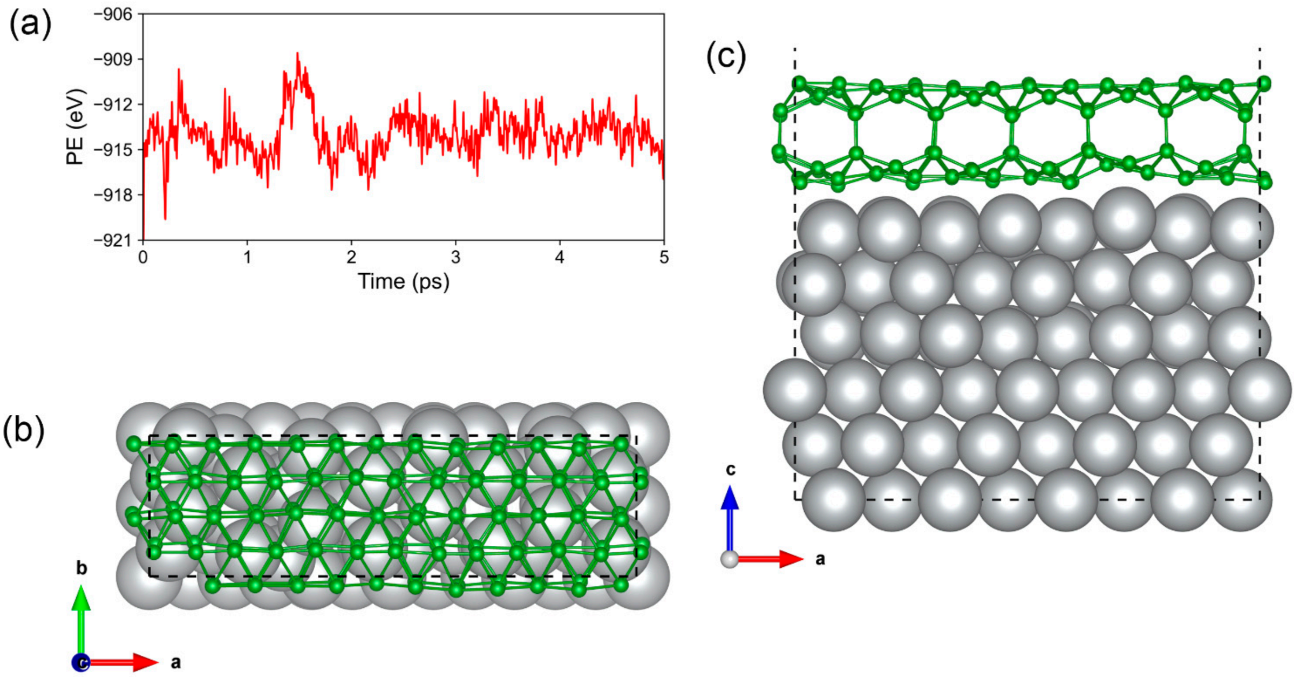

Figure S3. (a) The total potential energy fluctuation of BL-δ_6 during AIMD simulation at 500K. (b) The top view and (c) side view of the atomic configuration of BL-δ_6 on Ag (111) surface after 5 ps of AIMD simulation at 500 K.

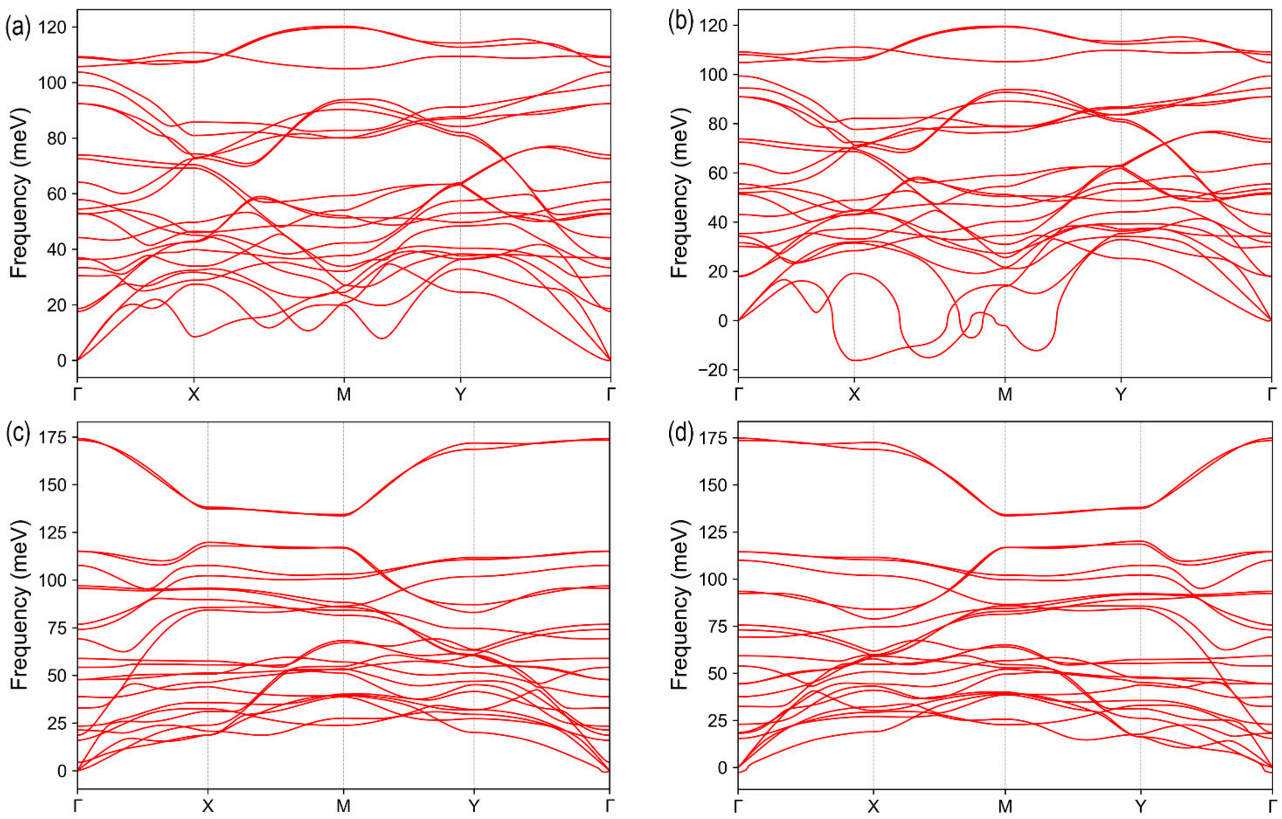

Figure S4. The phonon spectra of strains under (a) 13% and (b) 14% along **a** axis, (c) 8 % and (d) 9% along **b** axis.

Supplementary Note 2:

Here, elastic constants are obtained through fitting the strain and energy relationship for rectangular cell:[11, 12]

$$E_s = \frac{1}{2}C_{11}\varepsilon_{aa}^2 + \frac{1}{2}C_{22}\varepsilon_{bb}^2 + C_{12}\varepsilon_{aa}\varepsilon_{bb} + 2C_{44}\varepsilon_{ab}^2 \quad (1)$$

where E_s is the strain energy per unit area, ε_{aa}, ε_{bb} and ε_{ab} are the small axial strains along the ***a*** and ***b*** directions and the shear strain, respectively (Figure S4). The in-plane Young's modulus Y and Poisson ratio ν along an arbitrary direction can be calculated with the elastic constants as follows:

$$Y(\theta) = \frac{\Delta}{C_{11}s^4 + C_{22}c^4 + \left(\frac{\Delta}{C_{44}} - 2C_{12}\right)c^2 s^2} \tag{3}$$

$$\nu(\theta) = \frac{\left(C_{11} + C_{22} - \frac{\Delta}{C_{44}}\right)c^2 s^2 - C_{12}(c^4 + s^4)}{C_{11}s^4 + C_{22}c^4 + \left(\frac{\Delta}{C_{44}} - 2C_{12}\right)c^2 s^2} \tag{4}$$

where $\Delta = C_{11}C_{22} - C_{12}^2$, $c = \cos(\theta)$ and $s = \sin(\theta)$, θ is the angle relative to the ***a*** direction.

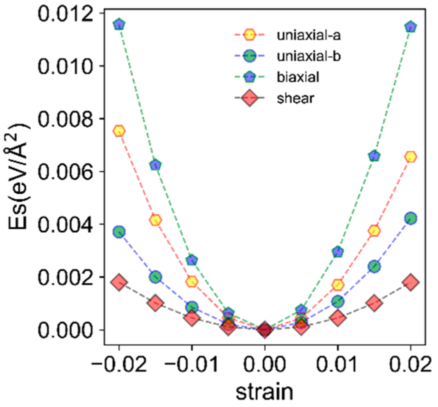

Figure S5. The strain energy per area under different kinds of strains for BL-δ_6.

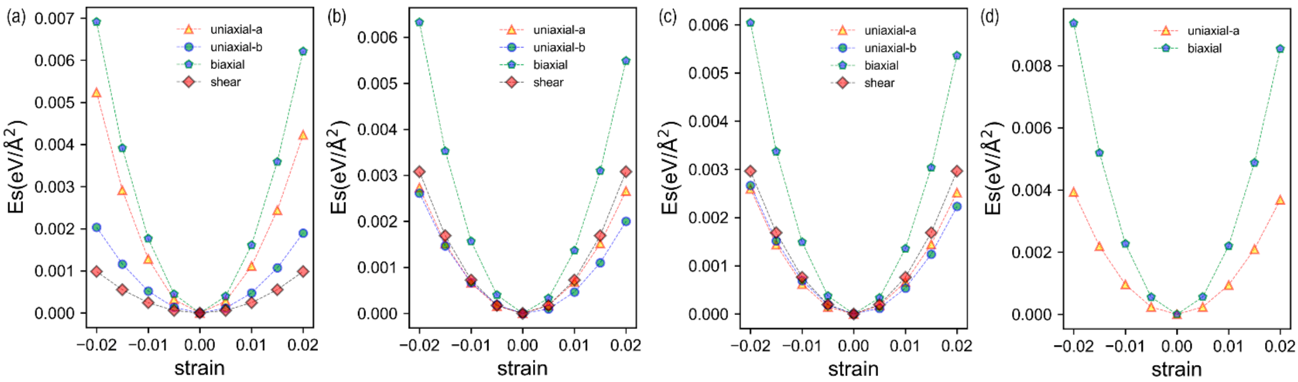

Figure S6. The strain energy per area under different kinds of strains for (a) δ_6, (b) β_{12}, (c) χ_3 and (d) graphene.

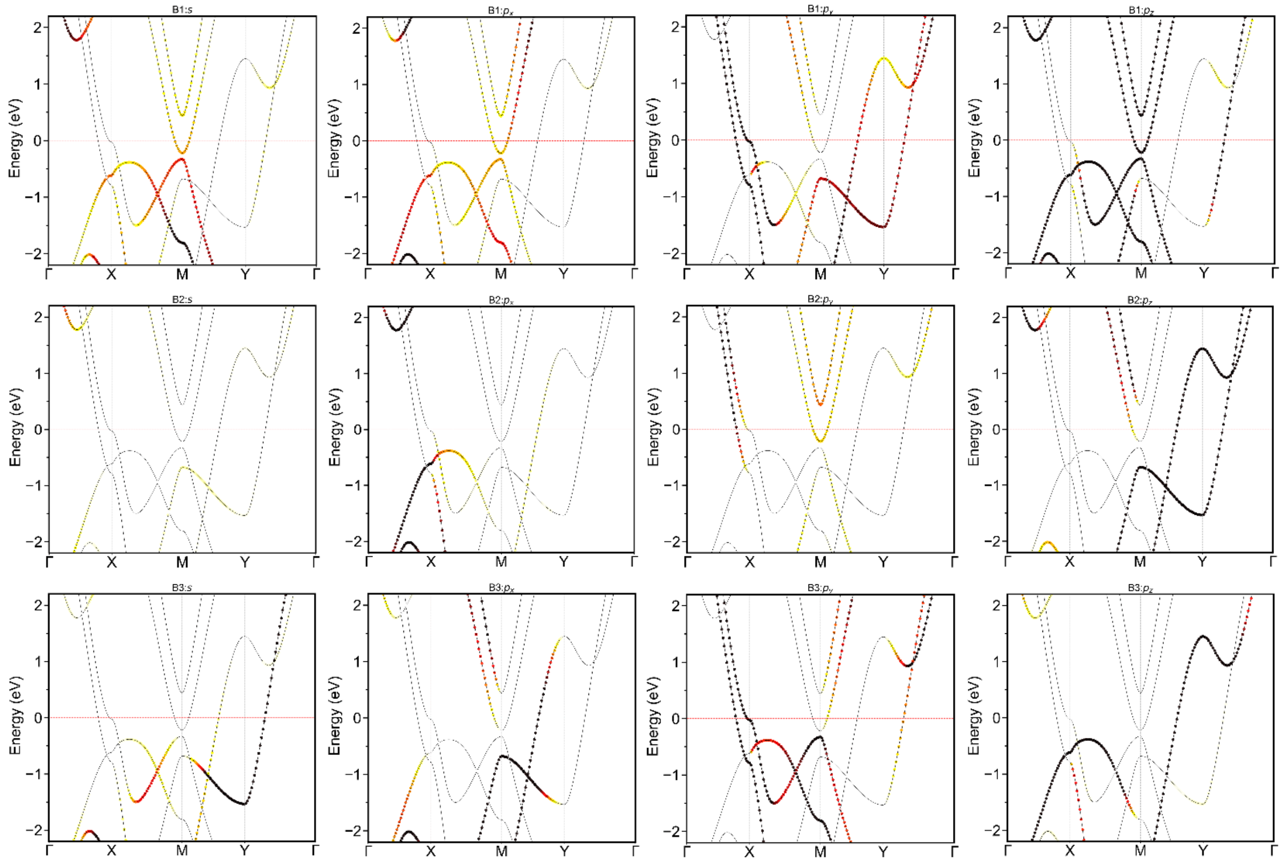

Figure S7. The atomic and orbital resolved band structures of BL-δ_6.

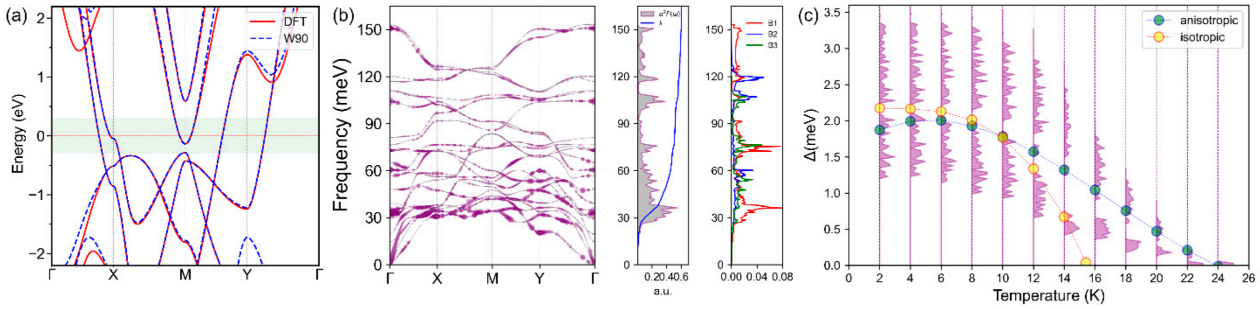

Figure S8. (a) The electronic band properties of BL-δ_6 under 3% tensile strain along **a** direction, red line and blue dashed line denote the results calculated from DFT and Wannier90, respectively. (b) Phonon band structure of BL-δ_6 weighted by EPC λ_{qv} with purple circles, isotropic Eliashberg function α^2F and EPC $\lambda(\omega)$, PHDOS from different kinds of boron atoms' contribution. (c) Evolution of the superconducting gap Δ_k as a function of temperature, calculated by solving the ME equations in the isotropic approximation (yellow dots and dashed line interpolation) and with a fully anisotropic solution where the purple shadowed regions indicate the magnitude distribution of the Δ_k and the light green dots connected with dashed line represents the average value of the entire anisotropic Δ_k.

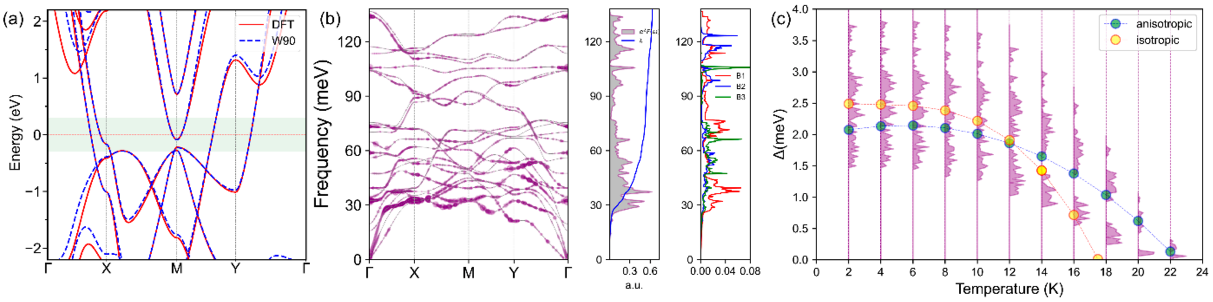

Figure S9. The results of BL-δ_6 under 6% tensile strain along **a** direction. The meaning of (a-c) are the same as Figure S8.

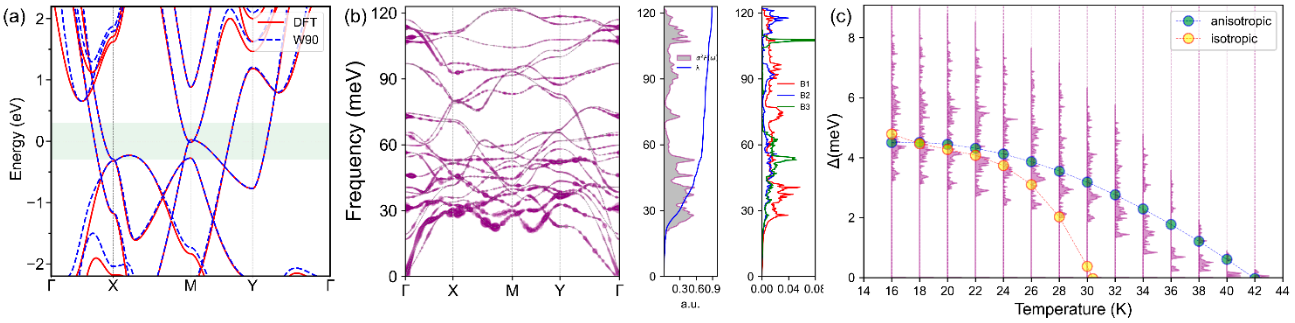

Figure S10. The results of BL-δ_6 under 10 % tensile strain along **a** direction. The meaning of (a-c) are the same as Figure S8.

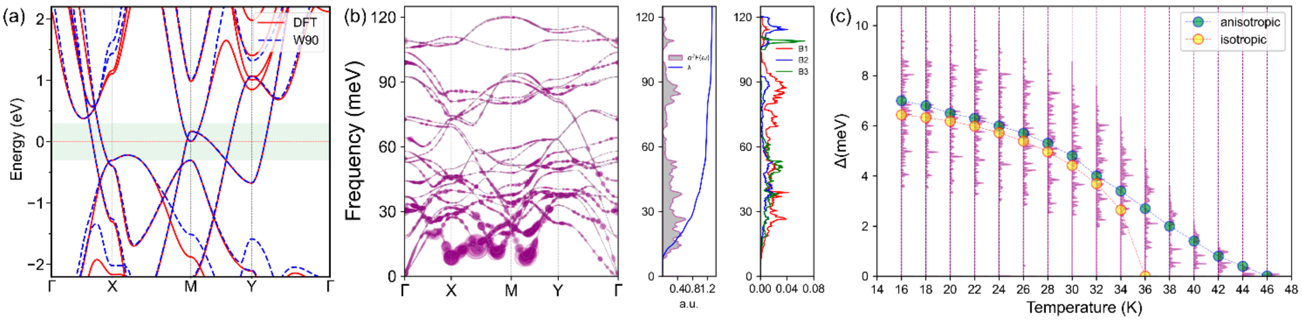

Figure S11. The results of BL-δ_6 under 13% tensile strain along **a** direction. The meaning of (a-c) are the same as Figure S8.

References:

(1) Kresse, G.; Furthmüller, J. Efficiency of ab-initio total energy calculations for metals and semiconductors using a plane-wave basis set. *Comp. Mater. Sci.* **1996,** *6*, 15-50.

(2) Kresse, G.; Furthmüller, J. Efficient iterative schemes for ab initio total-energy calculations using a plane-wave basis set. *Phys. Rev. B* **1996,** *54*, 11169-11186.

(3) Perdew, J. P.; Chevary, J. A.; Vosko, S. H.; Jackson, K. A.; Pederson, M. R.; Singh, D. J.; Fiolhais, C. Atoms, molecules, solids, and surfaces: Applications of the generalized gradient approximation for exchange and correlation. *Phys. Rev. B* **1992,** *46*, 6671-6687.

(4) Blöchl, P. E. Projector augmented-wave method. *Phys. Rev. B* **1994,** *50*, 17953-17979.

(5) Evans, D. J.; Holian, B. L. The Nose–Hoover thermostat. *J. Chem. Phys.* **1985,** *83*, 4069-4074.

(6) Nosé, S. A unified formulation of the constant temperature molecular dynamics methods. *J. Chem. Phys.* **1984,** *81*, 511-519.

(7) Giannozzi, P., et al. Advanced capabilities for materials modelling with Quantum ESPRESSO. *J. Phys.: Condens. Matter* **2017,** *29*, 465901.

(8) Baroni, S.; de Gironcoli, S.; Dal Corso, A.; Giannozzi, P. Phonons and related crystal properties from density-functional perturbation theory. *Rev. Mod. Phys.* **2001,** *73*, 515-562.

(9) Noffsinger, J.; Giustino, F.; Malone, B. D.; Park, C.-H.; Louie, S. G.; Cohen, M. L. EPW: A program for calculating the electron–phonon coupling using maximally localized Wannier functions. *Comput. Phys. Commun.* **2010,** *181*, 2140-2148.

(10) Pizzi, G., et al. Wannier90 as a community code: new features and applications. *J. Phys.: Condens. Matter* **2020,** *32*, 165902.

(11) Zhong, C.; Wu, W.; He, J.; Ding, G.; Liu, Y.; Li, D.; Yang, S. A.; Zhang, G. Two-dimensional honeycomb borophene oxide: strong anisotropy and nodal loop transformation. *Nanoscale* **2019,** *11*, 2468-2475.

(12) Cadelano, E.; Palla, P. L.; Giordano, S.; Colombo, L. Elastic properties of hydrogenated graphene. *Phys. Rev. B* **2010,** *82*, 235414.
